# Effects of isometric training and R.I.C.E. treatment on the arm muscle performance of swimmers with elbow pain

**DOI:** 10.1038/s41598-024-54789-0

**Published:** 2024-02-27

**Authors:** Weihan Li, Maryam Hadizadeh, Ashril Yusof, Mohamed Nashrudin Naharudin

**Affiliations:** Faculty of Sports and Exercise Science, Universiti Malaya, 50603 Kuala Lumpur, Malaysia

**Keywords:** Elbow pain, Elite freestyle swimmer, sEMG, Muscle recruitment velocity, MVC (muscle voluntary contract), Isometric training, R.I.C.E. treatment, Health services, Occupational health

## Abstract

The effects of IT and R.I.C.E. treatment on arm muscle performance in overhead athletes with elbow pain (EP) have been partially validated. However, there is a lack of research evidence regarding the efficacy of these two methods on arm muscle performance among swimmers with EP. The aim of this study was to investigate the trends and differences in the effects of IT and R.I.C.E. treatment on arm muscle performance among swimmers with EP. The main outcomes were the time effects and group effects of interventions on muscle voluntary contraction (MVC). Sixty elite freestyle swimmers from Tianjin, China, voluntarily participated in the study and completed a 10-week intervention program. Swimmers with EP in the IT group showed a positive trend in MVC, with an approximately 2% increase, whereas the MVC of subjects in the R.I.C.E. treatment group and control group decreased by approximately 4% and 5%, respectively. In comparison, the effects of the IT intervention on the MVC of the triceps and brachioradialis muscles in swimmers with EP were significant (p = 0.042 < 0.05, p = 0.027 < 0.05). The mean MVC value of the IT group (0.60) was greater than that of the other two groups (0.51, 0.50). IT has a beneficial impact on the MVC performance of the triceps and brachioradialis muscles in swimmers with EP. It is recommended that professionals consider incorporating IT into regular training routines to mitigate the risk of EP issues. Future research should examine the effectiveness of both interventions on hand-grip strength and completion time in 50-m freestyle swim drills in order for swimmers with EP to return to this sport.

## Introduction

Based on the kinematic characteristics of elbow joint movement in swimmers, the muscle voluntary contraction (MVC) of four major arm muscles (the biceps brachii, the triceps brachii, the brachioradialis, and the forearm flexors and extensors) determines the magnitude of propulsion force generated^1–4^. When swimmers suffer from elbow pain (EP), it inevitably has a negative impact on the voluntary contraction of their arm muscles^5,6^. While there are various methods used in sports rehabilitation and sports medicine to address EP problems, clinical surgeries and single-structure traction therapies, for instance, are associated with long treatment periods and high costs^7–9^. Moreover, topical ointment raises concerns about compliance with anti-doping regulations for swimmers^10,11^. These potential uncertainties and drawbacks increase the risk of premature career termination for swimmers with EP. Considering the aim of restoring the arm muscle MVC capacity of swimmers with EP rather than merely curing EP issues, employing a ‘sports prescription’ approach to enhance arm muscle MVC during training could be an effective method to facilitate the recovery of arm muscle MVC performance and expedite the return of swimmers with EP to their sport^12,13^. As potential intervention methods from ‘sports prescriptions’ in daily training conditions for EP swimmers, isometric training (IT) and rest, ice, compression, and elevation (R.I.C.E.) treatment have been proven to be feasible in a few studies^14,15^. However, the evidence supporting their effectiveness is insufficient.

The method of using surface electromyography (sEMG) tools to evaluate muscle performance is well established^16–20^. sEMG can accurately measure the segmental muscle performance of the human body without being limited by the use of situations such as underwater activities^21,22^, clinical treatment^23^, film and music performance^24^, and construction^20^. Its advantages include the following: the subjects will not experience limb wounds due to the use of implantable EMG tools, the method of measurement is flexible, and the measurement duration is adjustable^16,18^. According to previous relevant studies, MVCs have been widely discussed as key factors influencing arm muscle performance^25–27^. They are considered the gold standard indicators for assessing arm muscle functional capacity^27,28^ and are commonly employed to gauge changes brought about by interventions using amplitude trends, which aim to determine whether the impact is positive or negative^29,30^. EP issues have been shown to negatively affect MVC in swimmers, indirectly leading to disruptions or declines in arm muscle functional capacity and ultimately impacting personal best (PB) performance (as more than 80% of propulsion in swimming comes from upper limb muscle strength)^31–38^. Scholars have proposed various solutions to address this issue, but their effectiveness has been unsatisfactory for EP patients. Surgery can only restore daily activities for individuals with impaired mobility; however, their return to training or competitive levels requires additional expenses for rehabilitation, and the duration of recovery is uncertain, which often leads to the premature termination of athletic careers^39–41^. Oral medications, topical creams, or injected biologics may temporarily suppress EP, but it cannot be guaranteed that these substances comply with anti-doping regulations, which could disqualify athletes from competition and cause unrecognized physiological harm^42–46^. Furthermore, single-modality physical therapy, such as traction, can alter the muscle fibre length and radius to some extent, which can potentially result in a decrease in muscle functional capacity, creating a vicious cycle for EP patients during the process of recovery and return to the sport^47–49^. Therefore, measuring arm muscle performance in swimmers with EP by using sEMG tools, with MVC as the primary parameter, is a reliable research approach that can be used to evaluate the effectiveness of the interventional method and duration of IT and R.I.C.E. treatment and their impact on the recovery of arm muscle performance in swimmers with EP.

In summary, this study is the first to use sEMG to measure the performance of the arm muscles of swimmers with EP. The primary objective of this study was to investigate the amplitude trend in the MVC of the arm muscles of the biceps brachii, triceps brachii, brachioradialis, and forearm flexor and extensor of swimmers with EP before and after intervention. The secondary objective was to calculate and compare the effects of the intervention on the muscle MVC of the biceps brachii, triceps brachii, brachioradialis, and forearm flexor and extensor muscles in swimmers with EP. The main outcomes were the effects of time and intervention type on the MVC.

## Materials and methods

The study received ethical approval from the Universiti Malaya Ethics Approvals Sub-Committee, with reference number UM. TNC2/UMREC_1951. All participants signed informed consent before the study commenced (Supplementary Appendix 1, 2). All the experiments were performed in accordance with the University Malaya Research Ethics Guidelines (https://umresearch.um.edu.my/research-ethics/). The study was conducted from October 2022 to January 2023 at the Tianjin Evergrande Olympic Aquatic Sports Center, China.

### Sample size calculation

PASS 15 software was used as the main tool to calculate the sample size a priori in this study. The mean standard deviation results of the right arm muscle recruitment velocity (unit = milliseconds/ms) in 3 EP subjects after 5 weeks of intervention from the pilot experiment were indexed and inputted with the following statistical preconditions: the bilateral test α was 0.05*,* and the power was 0.90^50,51^. One-way analysis of variance (ANOVA) F tests were used to calculate the minimum number of subjects in each group, which was 15. The total sample of 45 subjects achieved 92% power to detect differences among the means versus the alternative of equal means using an F test with a 0.05 significance level. Considering that the rate of dropout is 20%^52^, the dropout-inflated enrolment sample size in each group is 19, and the total expected number of subjects was 57 (Supplementary Appendix 3).

### Subject recruitment

According to the calculated sample size, 60 right-handed elite freestyle swimmers (n = 60) were included in this study. The participants were all recruited from a professional swimming team, and the participants included 7 females and 53 males with EP who had more than 2 weeks of EP history in the anterior position before receiving the IT and R.I.C.E. interventions in this study. A chi-square test was conducted to examine the gender distribution among the 60 participants, and the results indicated a p value greater than 0.05 (p > 0.05). This suggests that there was no statistically significant difference in gender among the participants. Therefore, there was no separate analysis conducted for male and female participants in this study. The details of the sample characteristics are shown in Table 1. The details of the inclusion and exclusion criteria are as follows.

**Table 1:** Sample characteristics.

Characteristics	Healthy	Slight elbow pain	Critical elbow pain
Age (years)^a^	19.41 (0.62)	19.87 (0.92)	20.34 (0.34)
Height (cm)^b^	180.24 (2.98)	179.88 (3.41)	182.05 (2.94)
Weight (kg)^c^	68.77 (2.99)	69.91 (3.28)	70.22 (2.99)
Average weekly training hours^d^	25.04 (2.91)	26.17 (3.05)	25.00 (1.04)
Number of subjects^e^	18	21	21

Data are rounded to two decimal places.

^a–d^All values are presented as the mean (SD).

^e^All values are presented as numbers.

#### Inclusion criteria

- Achieved the title of elite swimmer in freestyle swimming (identified as a personal best (PB) time on 50 m freestyle swimming under 24.50 s for males and under 27.20 s for females) was achieved^53^.
- Age from 18 to 28 years; right-handed athletes.
- Subjects completed the assessment of the Body Parts Disability Evaluation form (BPDE) and Upper Limb Function Evaluation form (ULFE).
- Subjects graded as having slight pain, critical pain, and health conditions in both the BPDE and the ULFE assessments.

#### Exclusion criteria

- Participation in the pilot experiment.
- Paralysed swimming athlete.
- Female subjects in their menstrual period.
- Athletes receiving any form of physical therapy to prepare for competition and/or retirement.
- Athletes who in the past six months had suffered any injuries, such as fractures or impingement, or had undergone surgery or physical therapy.

### Identification of isometric training (IT) and R.I.C.E treatment

IT was found to be effective at improving pain and disability in patients with lateral elbow tendinopathy^54^, and a complete closed loop of IT training was used as an intervention method in this study. The closed loop was a 4-phase training session, which included warm-up, IT exercise, cooling-down, and rest and assessment. The warm-up phase included 5 min of forearm muscle stretching, 20 min of Bobi jumps, and 2 min of nonloaded rope skipping. The IT exercise included 3 × 30 barbell kneeled pronated-supinated isometrics and 3 × 30 lightweight seated neutral grips. The cooling-down phase included 5 min of forearm muscle stretching and 5 min of jogging. The last phase of rest and assessment is the subjects’ feedback on completing the intervention. Subjects who completed these four phases were regarded as complete sessions. Each session took approximately 30 min. The test was repeated 3 times a week, with an interval of 2 days between each repetition. Flowchart 1 shows the complete IT training closed loop.

**Flowchart 1:**
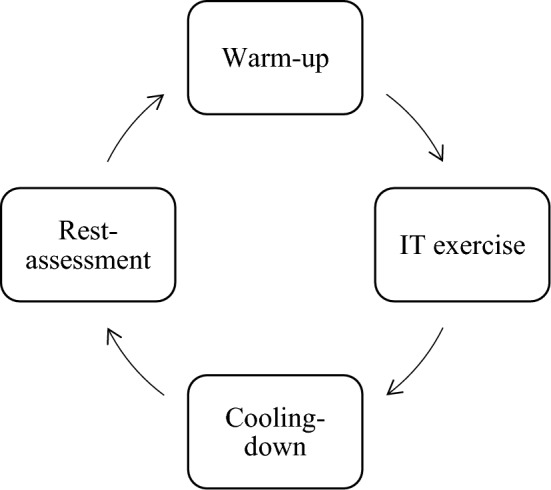
Complete IT training closed loop.

According to the existing R.I.C.E. treatment protocol^1,7,55^, subjects are required to be treated three times a week, with an interval of 2 days each time. The duration of each treatment was approximately 30 min. Flowchart 2 shows the process of the R.I.C.E. treatment protocol used in this study.

**Flowchart 2:**
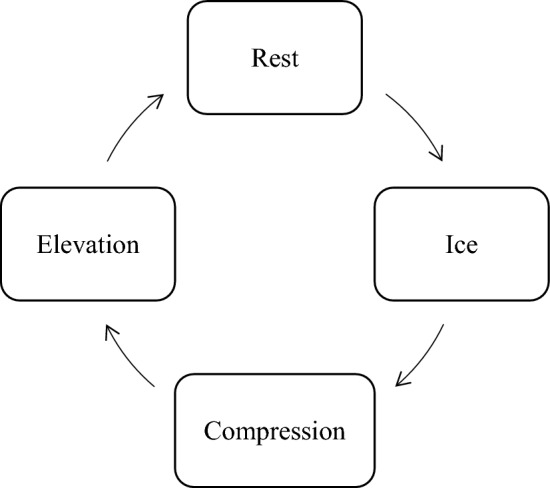
The R.I.C.E. treatment protocol.

### Subject grouping design

Based on the objectives, potential intervention methods, and participant characteristics of this study, 60 subjects were randomly divided into 3 groups. Group 1 served as the control group, in which the training routine of the subjects was maintained. The other 2 groups were intervention groups that received IT and R.I.C.E. treatment, respectively. Each group consisted of 6 healthy subjects, 7 EP patients with slight pain, and 7 EP patients with critical pain. The entire intervention period lasted for 10 weeks (a total of 50 days, with weekends as rest days). Subjects in the intervention groups had 1 h per day dedicated to the intervention programs under the supervision of the researcher WEIHAN to minimize the risk of unknown exercise-related injuries or exacerbation of EP issues. The data collection milestones were set as the first day on which the intervention began (week 1), the fifth week after the intervention started (week 5), and the day immediately following the intervention’s completion (week 10). Table 2 shows the details of the subjects’ grouping.

**Table 2:** Subject grouping.

Groups	Subjects	Number
Control Keep the Training Routine (KTR)	Healthy	6
	Slight pain	7
	Critical pain	7
Intervention Isometric Training (IT)	Healthy	6
	Slight pain	7
	Critical pain	7
Intervention R.I.C.E. treatment	Healthy	6
	Slight pain	7
	Critical pain	7
Total		60

### Measurement methods

**Figure 1:**
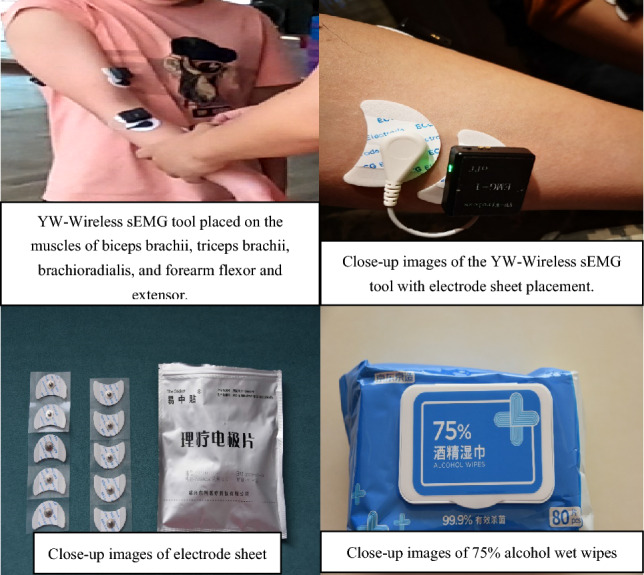
The specific steps for placing the YW-wireless sEMG tool.

According to the explanation of the main outcomes in this study, the parameters of arm-muscle voluntary contraction (MVC) for each subject group were measured by sEMG tools. The YW-Wireless tool was used to collect the parameters in the dryland environment (Supplementary Appendix 4). The specific steps were as follows: First, a 75% alcohol wipe was used to clean the biceps brachii, triceps brachii, brachioradialis, and forearm flexor and extensor muscles to reduce the interference signal generated by the cuticle and oil. Second, electrode patches were placed at the belly of the four muscles (Fig. 1).

**Figure 2:**
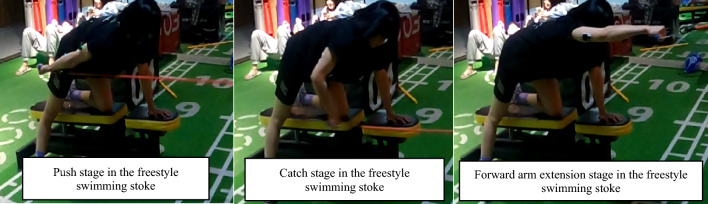
A 60-s simulated motion of the freestyle swimming stroke technique with the YW-wireless sEMG tool.

In a dryland environment, the subject was asked to perform a 60-s simulated motion of the freestyle swimming stroke technique (Fig. 2). The subject was shown to imitate three motion phases on a bench with elastic resistance bands, which included entering (forward arm extension), catching (elbow flexion and external rotation), and pushing (rearward arm extension). The strength is adjusted according to the resistance of the elastic resistance band used by the subject, and the longer/tighter the elastic resistance band is stretched, the closer the test subject is to approaching the maximum exertion load^56^. The regulation principle was that the subjects should not experience any elbow pain when performing the simulated movements.

### Methods of data processing and analysis

MATLAB software (version R2021b) was used as the main tool for processing and extracting the raw sEMG data. The Daubechies ‘db4’ wavelet transformation^57^ was employed to preprocess the raw sEMG signals for filtering oversize or abnormal noise data. The compressed sensing (CS) method^58^ was used to filter and extract the sEMG envelope value for calculating the muscle recruitment velocity of the subject’s arm muscle, after which the muscle voluntary contraction (MVC) value was computed. For the specific sEMG signal data, relevant value identification standards and formulas, please refer to Supplementary Appendix 5. Before conducting the data analysis, all the data were subjected to a normality distribution test via the Shapiro‒Wilk test^59^ on the entire dataset. The p values for all the data at each time point were greater than 0.05 (p > 0.05), confirming a normal distribution (Supplementary Appendix 6).

Repeated measures two-way analysis of variance (ANOVA) was used to examine the effectiveness in terms of the group effects (IT and R.I.C.E. treatment, p_group effects_ < 0.05) and time effects (intervention duration from 1 to 10 weeks, p_time effects_ < 0.05) on the MVC of the participants^60^. If the interaction between the intervention methods and period had a statistically significant impact on MVC, with a p value less than 0.05 (p_interaction_ < 0.05), further individual tests were conducted to test the effects of both group and time separately^61^. If the interaction effect was not significant, the main effects were analysed^62^. Post hoc tests were conducted on the results of the two-way ANOVA to determine the authenticity and to reduce the likelihood of false-positive results^63^.

### Study procedures

**Figure 3:**
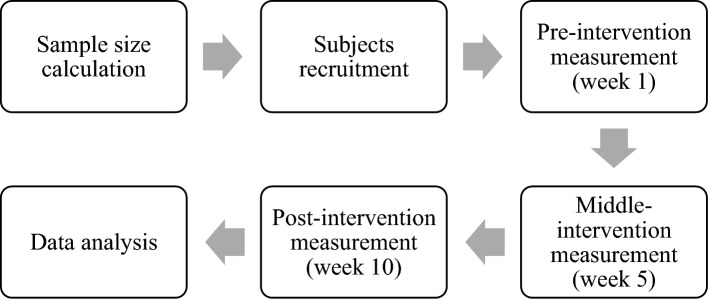
The steps of this study procedure.

Based on the description in the methods and materials section, this study consists of 6 steps. First, the sample size was calculated, and eligible subjects were recruited based on the predetermined inclusion and exclusion criteria. Then, data corresponding to the potential intervention periods were collected at weeks 1, 5, and 10. Finally, the collected data were analysed. Figure 3 shows the steps of this study procedure.

### Institutional review board statement

UM Research Ethical Approve reference number UM. TNC2/UMREC_1951.

## Results

### MVC amplitude trend

The trend in the amplitude of the MVC in each group at each time point was calculated. Table 3 shows that the average MVC in the IT group exhibited the greatest increase of approximately 2% in the group’s average amplitude trend from the first week to the tenth week. The MVC results of the R.I.C.E. treatment group were consistent from the first week to the tenth week but decreased by approximately 4% in the fifth week. The control group showed a decreasing trend of approximately 5%.

**Table 3:** MVC amplitude trend.

Groups	Time point	BB^a^	TB^b^	BM^c^	FFE^d^	Average^e^
Control	1st week	0.28	0.39	0.34	0.49	0.38
	5th week	0.31	0.39	0.30	0.36	0.34
	10th week	0.33	0.33	0.24	0.42	0.33
		+ 0.05	− 0.06	− 0.1	− 0.07	− 0.05
R.I.C.E treatment	1st week	0.39	0.40	0.38	0.40	0.39
	5th week	0.33	0.28	0.42	0.36	0.35
	10th week	0.35	0.51	0.29	0.41	0.39
		− 0.04	+ 0.11	− 0.09	+ 0.01	None
IT	1st week	0.43	0.46	0.49	0.41	0.45
	5th week	0.38	0.54	0.52	0.44	0.47
	10th week	0.47	0.59	0.32	0.49	0.47
		+ 0.04	+ 0.13	− 0.17	+ 0.08	+ 0.02

All values are rounded to two places.

The average represents the average MVC amplitude of the 4 muscles.

*BB* biceps brachii muscle, *TB* triceps brachii muscle, *BM* brachioradialis muscle, *FFE* forearm flexor and extensor muscles.

^a–e^All values are presented as numbers.

In detail, for the arm muscles, the percentage amplitude of the MVC in the FFE decreased by approximately 7% from week 1 to week 10 in the control group; the percentage amplitude of the MVC in the R.I.C.E. treatment group increased by approximately 1%, and that of the IT group increased by approximately 8%. The percentage amplitude of MVC in the BM decreased with time and was decreased by approximately 10%, 9% and 17% in the control group, R.I.C.E. treatment group and IT group, respectively, at week 10. The MVC of the TB in the control group showed a decreasing trend of approximately 6% and increased by approximately 11% and 13%, respectively, in both the R.I.C.E. treatment and IT groups. The BB in both the control and IT groups showed increasing trends of approximately 5% and 4%, respectively, and in the R.I.C.E. treatment showed a decreasing trend of approximately 4%.

### Differences in the MVC of the arm muscles

Two-way repeated-measures ANOVA was used to analyse the effects of intervention time on arm muscle MVC across all groups (Table 4). The results for the biceps brachii showed that the data met the assumption of sphericity^64^. The interaction effect between group and intervention time was tested (F = 1.429, p = 0.229 > 0.05, and η^2^ = 0.048), which indicated that the interaction effect between group and intervention time had no statistically significant impact on the biceps brachii muscle. The main effects of intervention time and group were tested, respectively resulting in F = 1.992, p = 0.141 > 0.05, η^2^ = 0.034, and F = 0.891, p = 0.416 > 0.05, η^2^ = 0.030. These findings suggested that intervention time and group had no statistically significant impact on the biceps brachii muscle.

**Table 4:** Time and group effects.

Muscles	Groups	Week 1^a^	Week 5^b^	Week 10^c^	F_interaction_/p_interaction_/η^2^	F_time_/p_time_/η^2^	F_group_/p_group_/η^2^
BB (biceps brachii)	Control	0.41 ± 0.16	0.42 ± 0.14	0.47 ± 0.21	1.429/0.229/0.048	1.992/0.141/0.034	0.891/0.416/0.030
	R.I.C.E. treatment	0.5 ± 0.19	0.44 ± 0.21	0.44 ± 0.22			
	IT	0.48 ± 0.13	0.45 ± 0.15	0.55 ± 0.21			
TB (triceps brachii)	Control	0.55 ± 0.2	0.49 ± 0.18	0.45 ± 0.22	1.657/0.165/0.055	0.950/0.390/0.016	3.344/0.042/0.105
	R.I.C.E. treatment	0.52 ± 0.26	0.46 ± 0.23	0.54 ± 0.2			
	IT	0.57 ± 0.17	0.58 ± 0.2	0.66 ± 0.2			
BM (brachioradialis)	Control	0.43 ± 0.15	0.43 ± 0.18	0.36 ± 0.17	0.979/0.422/0.033	2.495/0.087/0.042	3.845/0.027/0.119
	R.I.C.E. treatment	0.48 ± 0.15	0.51 ± 0.2	0.5 ± 0.25			
	IT	0.56 ± 0.18	0.56 ± 0.18	0.45 ± 0.23			
FFE (forearm flexor and extensor)	Control	0.54 ± 0.14	0.51 ± 0.27	0.47 ± 0.21	1.537/0.196/0.051	0.405/0.668/0.007	0.277/0.759/0.010
	R.I.C.E. treatment	0.57 ± 0.24	0.49 ± 0.2	0.47 ± 0.19			
	IT	0.49 ± 0.16	0.55 ± 0.2	0.57 ± 0.2			
Average (4 muscles)	Control	0.48 ± 0.12	0.46 ± 0.16	0.44 ± 0.16	0.929/0.450/0.032	0.512/0.600/0.009	2.199/0.120/0.072
	R.I.C.E. treatment	0.52 ± 0.15	0.47 ± 0.16	0.49 ± 0.16			
	IT	0.53 ± 0.11	0.54 ± 0.12	0.56 ± 0.16			

F, p, & η^2^ All values are rounded to three places.

^a–c^All values are presented as the mean value of the MVC percentage.

^a–c^All values are rounded to two places.

The results for the triceps brachii muscle showed that the data met the assumption of sphericity. The interaction effect between group and intervention time was tested, resulting in F = 1.657, p = 0.165 > 0.05, and η^2^ = 0.055. This finding suggested that the interaction effect between group and intervention time had no statistically significant impact on the triceps brachii muscle. The main effect of intervention time was tested, resulting in F = 0.950, p = 0.390 > 0.05, and η^2^ = 0.016. These findings indicated that intervention time had no statistically significant impact on the triceps brachii muscle. However, the main effect of grouping was tested, with F = 3.344, p = 0.042 < 0.05, and η^2^ = 0.105. This finding indicated that the grouping had a statistically significant impact on the triceps brachii muscle, explaining 10.5% of the variance. Furthermore, least significant difference (LSD) comparisons revealed statistically significant differences between the control group and the IT group (p = 0.020 < 0.05) and between the R.I.C.E. treatment group and IT group (p = 0.045 < 0.05); moreover, in the specific difference comparison, the average value during intervention in the IT group (0.60) was higher than that in the R.I.C.E. treatment group (0.51).

The results for the brachioradialis muscle showed that the data met the assumption of sphericity. The interaction effect between group and intervention time was tested, resulting in F = 0.979, p = 0.422 > 0.05, and η^2^ = 0.033. This finding indicated that the interaction effect between group and intervention time had no statistically significant impact on the brachioradialis muscle. The main effect of intervention time was tested, resulting in F = 2.495, p = 0.087 > 0.05, and η^2^ = 0.042. This finding suggested that intervention time had no statistically significant impact on the brachioradialis muscle. However, the main effect of grouping was tested, resulting in F = 3.845, p = 0.027 < 0.05, and η^2^ = 0.119. This finding indicated that grouping had a statistically significant impact on the brachioradialis muscle, explaining 11.9% of the variance. Further LSD comparisons revealed a statistically significant difference between the control group and the IT group (p = 0.010 < 0.05), as did the specific difference comparison; the average value during the intervention in the IT group (0.52) was greater than that of the R.I.C.E. treatment group (0.50).

The results for the forearm flexor and extensor muscles showed that the data met the assumption of sphericity. The interaction effect between group and intervention time was tested, resulting in F = 1.537, p = 0.196 > 0.05, and η^2^ = 0.051. This finding suggested that the interaction effect between group and intervention time had no statistically significant impact on the forearm flexor or extensor muscles. The main effects of intervention time and group were tested, resulting in F = 0.405, p = 0.668 > 0.05, η^2^ = 0.007, and F = 0.277, p = 0.759 > 0.05, η^2^ = 0.010, respectively. These findings suggested that intervention time and group had no statistically significant impact on the forearm flexor or extensor muscles.

The results for an average of 4 muscles showed that the data met the assumption of sphericity. The interaction effect between group and intervention time was tested, resulting in F = 0.929, p = 0.450 > 0.05, and η^2^ = 0.032. This finding suggested that the interaction effect between group and intervention time had no statistically significant impact on the average of the 4 muscles. The main effects of intervention time and group were tested resulting in F = 0.512, p = 0.600 > 0.05, η^2^ = 0.009, and F = 2.199, p = 0.120 > 0.05, and η^2^ = 0.072, respectively. These results suggested that intervention time and group had no statistically significant impact on the average of the 4 muscles.

Post hoc tests were conducted on the aforementioned two-way ANOVA results to determine whether the observed differences were authentic and not due to chance^63,65^. The results indicated that the main effect of intervention time on the brachioradialis muscle was not statistically significant. However, when using least significant difference (LSD) tests, a statistically significant difference was found between Week 5 and Week 10. Similarly, regarding the average MVC amplitude of the 4 muscles, the main effect of grouping was not statistically significant, but the LSD comparisons revealed a statistically significant difference between the control and IT groups. These findings may be attributed to the accumulation of probability errors leading to false-positive results (Type I error)^63^. To address this issue, a more stringent Bonferroni correction method was applied^63,66^. The results in Table 5 showed that in the brachioradialis muscle, the data comparison between Week 5 and Week 10 yielded t = − 2.207, p = 0.093 > 0.05, and in an average of 4 muscles, the data comparison between the control and IT groups yielded t = 2.079, p = 0.124 > 0.05. Therefore, the above differences were confirmed to be false-positive results, and the remaining results from two-way ANOVA were true.

**Table 5:** Results of the post hoc tests.

Muscles	Groups	t_Group_/*p*_Group_	Weeks	t_Time_/*p*_Time_
BB	Control and R.I.C.E.	0.565/0.568	1 and 5 weeks	− 1.304/0.200
	Control and IT	1.326/0.189	1 and 10 weeks	0.778/0.447
	R.I.C.E. and IT	0.761/0.452	5 and 10 weeks	1.962/0.059
TB	Control and R.I.C.E.	0.348/0.732	1 and 5 weeks	− 1.086/0.285
	Control and IT	2.370/0.020	1 and 10 weeks	0.121/0.914
	R.I.C.E. and IT	2.022/0.045	5 and 10 weeks	1.273/0.205
BM	Control and R.I.C.E.	2.000/0.050	1 and 5 weeks	0.500/0.629
	Control and IT	2.651/0.010	1 and 10 weeks	− 1.613/0.119
	R.I.C.E. and IT	0.651/0.510	5 and 10 weeks	− 2.207/0.031
FFE	Control and R.I.C.E.	0.065/0.947	1 and 5 weeks	− 0.548/0.581
	Control and IT	0.674/0.502	1 and 10 weeks	− 0.906/0.365
	R.I.C.E. and IT	0.609/0.545	5 and 10 weeks	− 0.343/0.732
Average	Control and R.I.C.E.	0.868/0.390	1 and 5 weeks	− 1.000/0.338
	Control and IT	2.079/0.041	1 and 10 weeks	− 0.778/0.460
	R.I.C.E. and IT	1.211/0.277	5 and 10 weeks	0.222/0.825

All values are rounded to three places.

The FFE represents the forearm flexor and extensor muscles.

The average represents the average value of 4 muscles.

*BB* biceps brachii muscle, *TB* triceps brachii muscle, *BM* brachioradialis muscle.

## Discussion

This study provides evidence of the greater effectiveness of IT compared to R.I.C.E. treatment in influencing the difference in triceps brachii and brachioradialis MVC among swimmers with EP. The amplitude trend of the arm muscle MVC for swimmers with EP using IT as an intervention method exhibited a positive growth trend, with an approximate 2% increase. The remaining two groups of subjects showed a decreasing trend in arm muscle MVC. The results of the two-way ANOVA and post hoc tests demonstrated that all the data met the assumptions of sphericity and normality. The main effect test revealed that the group effect had a statistically significant impact on the MVC of the triceps brachii and brachioradialis muscles (p = 0.042 < 0.05, p = 0.027 < 0.05). Statistically significant differences were detected between the control group and the IT group in terms of the MVC of the triceps brachii (p = 0.020 < 0.05) and between the R.I.C.E. treatment group and the IT group in terms of the MVC of the triceps brachii (p = 0.045 < 0.05). In the specific difference comparison, the average value during the intervention of the IT group (0.60) was greater than that of the R.I.C.E. treatment group (0.51). Additionally, statistically significant differences were found between the control group and the IT group in terms of the MVC of the brachioradialis muscle (p = 0.010 < 0.05). In the specific difference comparison, the average value during the intervention in the IT group (0.52) was greater than that of the R.I.C.E. treatment groups (0.50).

The results of the trend in arm muscle MVC amplitudes indicate that IT is most effective for swimmers with EP (the average value in the MVC increased by 2% compared to that in the other two groups, p < 0.05). Specifically, in the TB and BM muscles, there was an increase of 13% (p < 0.05) and a decrease of 17% (p < 0.05), respectively. This suggests that IT has a direct impact on the autonomous contraction ability of TBs and BMs in swimmers with EP. The increase in MVC for TB indicates that IT improves muscle fibre thickness, allowing for a significant increase in TB contraction ability and leading to increased propulsion during the push stage of the freestyle swimming technique^54,67–70^. The direct effect of IT on the BM results in a decrease in its contraction ability, stabilizing the elbow joint during the catch stage in freestyle swimming stroke^71–73^. Additionally, the autonomous contraction ability of muscles is closely related to muscle electromyography (EMG) activity^22,74–77^. It remains unknown whether the IT-induced improvement in MVC for swimmers with EP also influences muscle EMG activity. However, this aspect is one of the keys focuses of our future research directions.

Although R.I.C.E. treatment led to improvements of 1% and 11%, respectively, in specific muscles of the MVC (forearm flexors and extensor and triceps brachii), these effects were not validated by the group-effect and time-effects analyses. This phenomenon might be attributed to the temporary relief of muscle discomfort caused by the reduction in blood flow due to the icing action from the R.I.C.E. treatment, which leads to a numbing effect in the affected area^1,78–81^. This leads to a transient alleviation of muscle discomfort, acting as a ‘placebo’ effect rather than a genuine improvement in the muscle’s functional capacity^82–84^. Therefore, the RICE treatment is more suitable for temporarily relieving pain rather than improving arm muscle performance. Furthermore, the results also demonstrated the growth trend of the triceps brachii (TB) and brachioradialis (BM) muscles in the IT group. This phenomenon could have a positive impact on the hand-grip strength of swimmers with EP^85–87^ and is closely related to arm muscles, such as the TB and BM, which are essential factors determining the functional capacity of the arm muscles^88–92^. Therefore, with the improvement in arm muscle MVC, hand-grip strength is also likely to be affected, which could indirectly influence the completion time of swimmers with EP during short-distance freestyle swimming, ultimately enabling swimmers with EP to return to their sport.

The result of this study suggests the effectiveness of IT compared to R.I.C.E. treatment in influencing the difference in triceps brachii and brachioradialis MVC among swimmers with EP. However, no group effects were observed in the other muscles (biceps brachii and forearm flexors and extensors), which suggests that individual biological differences, such as variations in height and weight, might lead to different responses from those of the IT^93–95^. This indicates that the absence of significant group effects is not due to IT having no effect on these muscles but highlights the need to address and minimize interindividual biological differences to further understand the impact of IT on the arm muscle MVC in EP patients. Moreover, no time effects were observed in the results, which indicates that the intervention period (10 weeks) might not be sufficient for significant improvement in arm muscle MVC for EP patients. This could be related to the high baseline levels (week 1) of arm muscle MVC in the participants^96,97^. According to the literature, baseball players with EP injury who have undergone surgical treatment achieve an 90% recovery of their MVC to healthy levels after a 6-week program of muscle strength rehabilitation^5,98^. Therefore, for these swimmers with EP, a longer intervention period might be required to observe differences in arm muscle MVC more easily at various time points.

## Limitations

Although the intervention methods used in this study successfully restored the muscle performance of swimmers with EP, we still do not know the deeper effects on muscle performance, such as the influence of muscle fibres. Therefore, the results of this study are limited to the expression of superficial muscle performance dimensions. Further research on deeper muscle performance may require the use of implantable EMG or probe measurements. In addition, we still do not know how long the effect of an intervention on swimmers with EP can be maintained. Therefore, the effective duration of intervention should be explored in future research to design appropriate training sessions for swimmers with EP.

## Conclusion

After completing the 10-week intervention experiment, swimmers with EP showed improvements in their arm muscle MVC performance. The use of IT as an intervention method resulted in the greatest improvement among EP patients. The group-effect analysis demonstrated the effectiveness of IT compared to R.I.C.E. treatment in influencing the difference in triceps brachii and brachioradialis MVC among swimmers with EP. However, limitations in this study are inevitable. The intervention period should be extended, and the physiological differences among individual samples should be minimized to facilitate easier observation of time effects. Future related research should investigate hand-grip power and completion time in short-distance freestyle swimming drills based on the effectiveness of improving arm muscle MVC in swimmers with EP to meet return-to-play demands.

### Supplementary Information

Supplementary Information.

## Data Availability

Raw and processed data are available to the corresponding author upon request.
