# Supplementary material for: Effects of isometric training and R.I.C.E. treatment on the arm muscle performance of swimmers with elbow pain
Source: Sci Rep. 2024 Feb 27;14:4736. doi: 10.1038/s41598-024-54789-0 (PMC10899567; doi:10.1038/s41598-024-54789-0)
Supplement: Supplementary file 1 — Supplementary Information. [file 41598_2024_54789_MOESM1_ESM.docx]

**Appendix 1:** Human Ethics Approval Letter


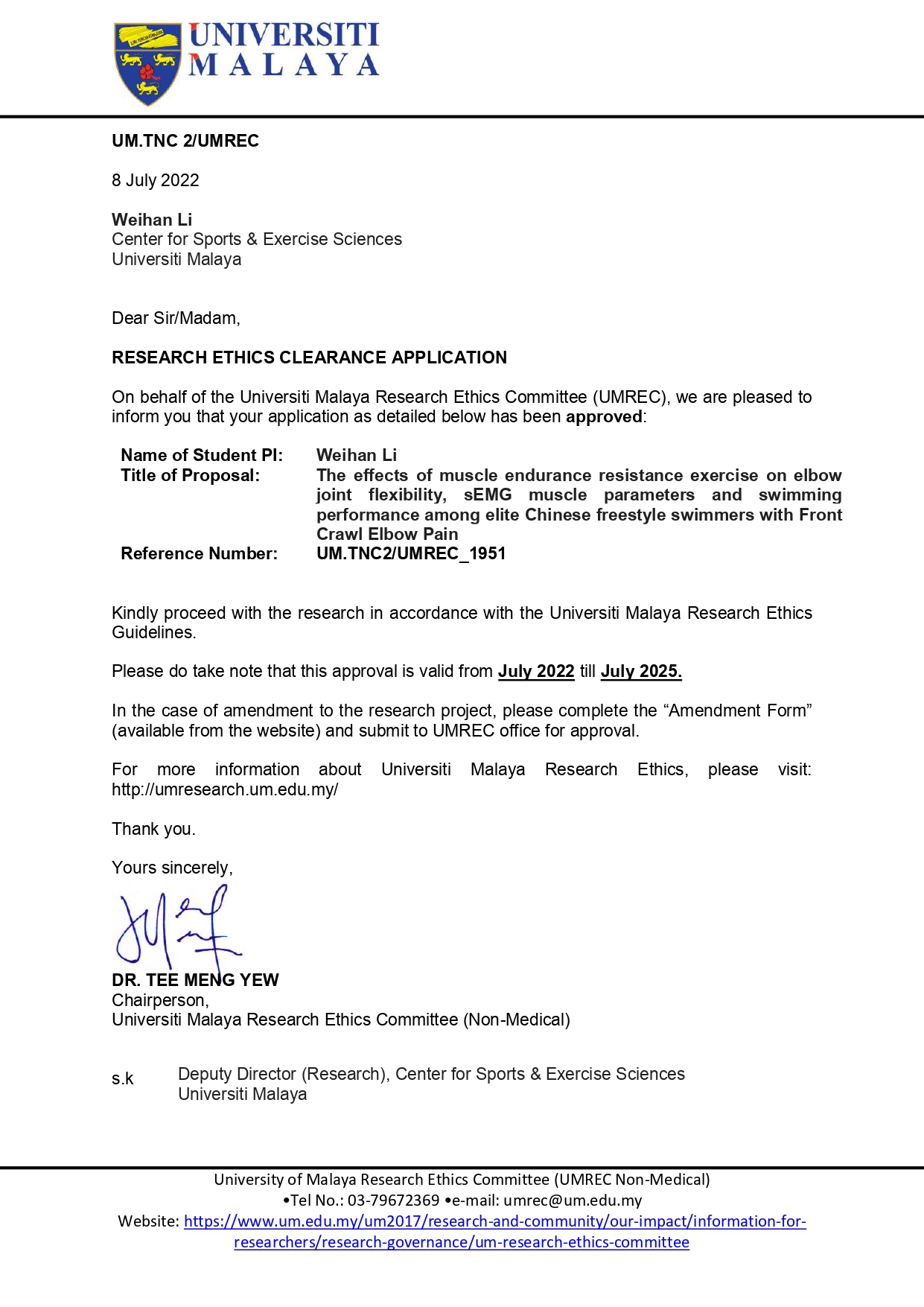


**Appendix 2:** Informed Consent Form


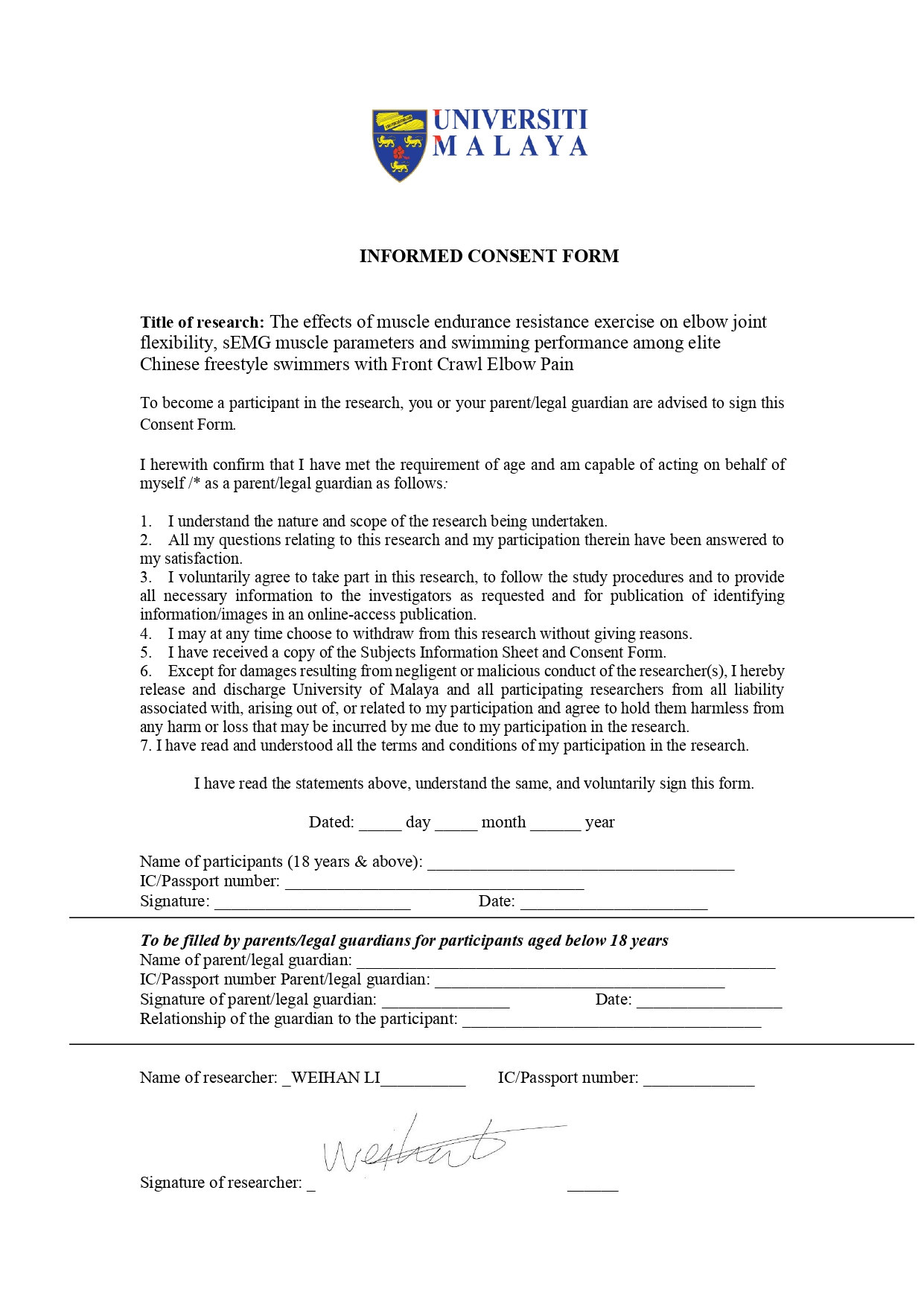


**Appendix 3:** sample size calculation


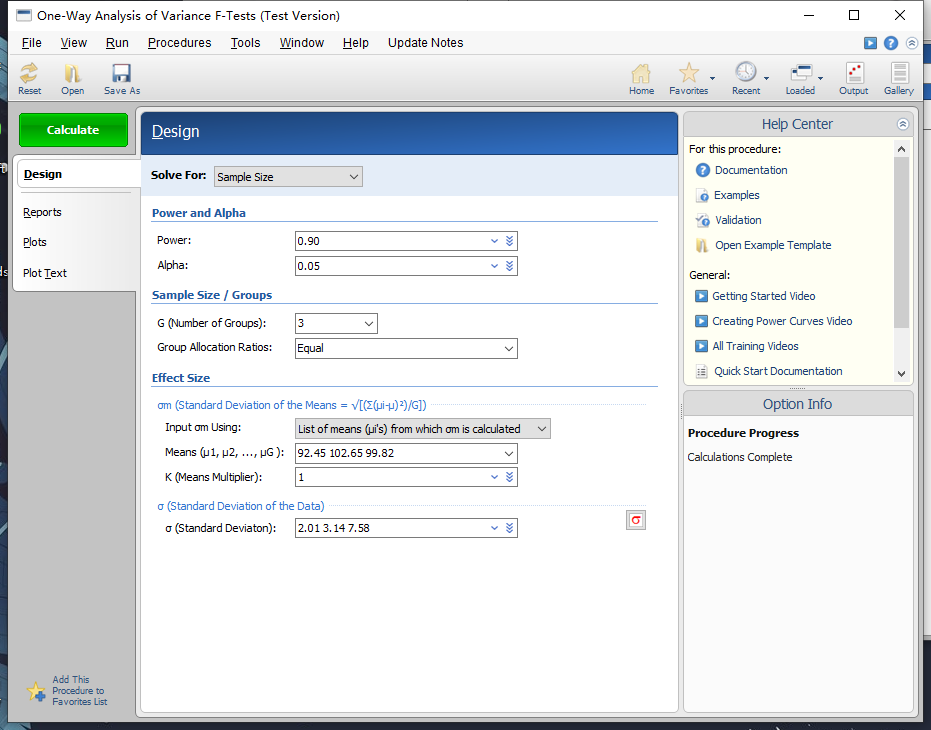


**Appendix 4:** Measurement tools and software

YW-Wireless is an EMG signal acquisition system consisting of a wireless EMG sensor, which is combined with a multi-channel data receiving box, acquisition, and analysis software. It can realize dynamic storage, synchronization with a 3D motion capture system, dynamometer, etc., and realize the overall analysis of sports biomechanics. The technical specifications are an input impedance of 10G ω; the ratio of common mode rejection (CMRR) is 130dB; sampling rate 1000Hz; the hardware gain is 1000 times, and the software gain is 500, 1000,2000,3000,4000,5000; bandwidth 7Hz-1000Hz; the signal input range is 0-10mV; wireless transmission 20 meters; wireless sensor size 34mm*28mm*11mm.


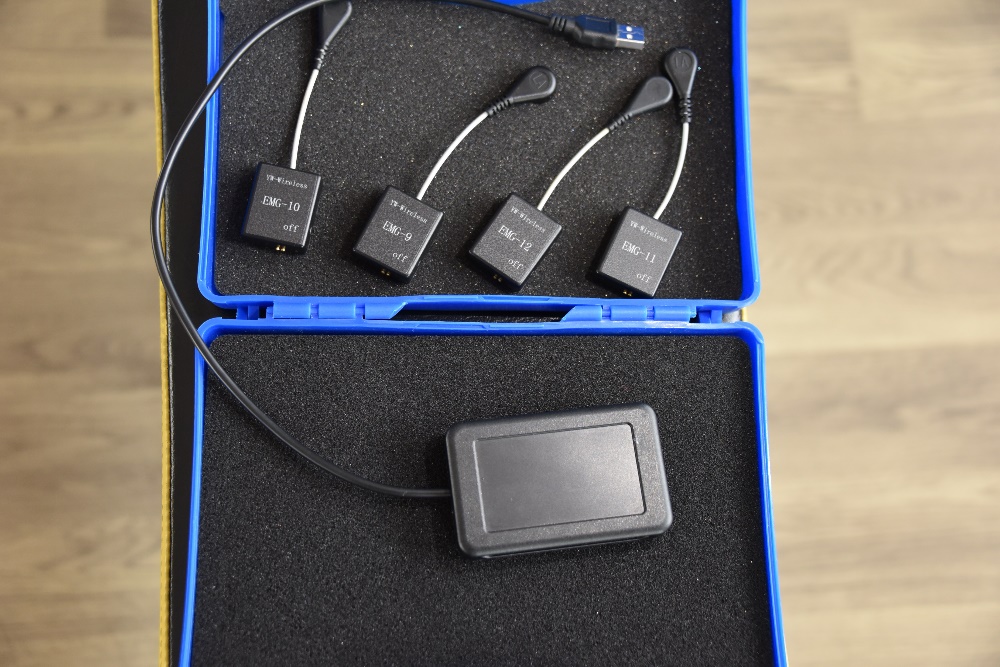


**Appendix 5:** The raw sEMG signal data process in MATLAB software.

The raw sEMG signal data were decomposed into 6 layers by ‘db4’ wavelet, and the scale coefficients from the second to sixth layers are reconstructed. The passband frequency is 7.8-125HZ. After the filtering, 3 subjects with oversize noise data were excluded.

The sEMG envelope value at the center of the window represented by using the absolute average value of sEMG signals in the sliding window with a length of 50 frames and a step size of 20 frames. According to the sEMG envelope signal, the peak value of sEMG signal is extracted to calculate the muscle recruitment velocity and MVC for the subjects’ arm muscles. The requirements for the peak value identification are: the maximum value is the peak, the relative height of the peak is greater than 100μV, and the peak interval is greater than 1.0s.

Identification of the muscle recruitment velocity is the time taken for the sEMG envelop peak signal to rise from 10% to 70% height. The calculation methods of muscle recruitment velocity is conducted that the starting point is set by the first time point below 10% height and the ending point is set by the point above 70% height in the signal section for each peak. Linear interpolation method was used to calculate the time require for sEMG envelop peak value from 10% to 70% height. To reduce the influence of peak value error identification on calculation, the results that made over-contribution value to the standard deviation (SD) of muscle recruitment velocity in a single measurement were discarded, which the SD of muscle recruitment velocity was less than 20ms and the averaged screening results were conducted. Therefore, the result of muscle recruitment velocity of each arm muscle from the measurement was obtained.

In the study, MVC (maximum voluntary contract) is the ratio of the average peak value in the right arm muscle recruitment velocity in each measurement to the peak value of the muscle recruitment velocity in the maximum exertion measurement. Based on this explanation, it normalized the average value of muscle recruitment velocity and the ratio of muscle recruitment velocity corresponding to the highest peak value, which obtained the MVC. Therefore, a calculation formula (1.1) is defined that the maximum voluntary contraction of the K^th^ subject is $\mathrm{MVC}\left( k \right)$, which ${MVC}_{(m,t)}^{(k)}$ indicates the maximal autonomic contractility of the M^th^ muscle of the K^th^ subject in the *t* week.

$$\mathrm{MVC}\left( k \right)=\left[ \begin{matrix} {MVC}_{(1,1)}^{(k)} & {MVC}_{(1,5)}^{(k)} & {MVC}_{(1,10)}^{(k)} \\ \vdots& \ddots& \vdots\\ {MVC}_{(4,1)}^{(k)} & {MVC}_{(4,5)}^{(k)} & {MVC}_{(4,10)}^{(k)} \end{matrix} \right]$$

***Formula 1.1***

The procedures of data analysis were following:

Start

The frequency domain filtering of ‘db4’ wavelet transform

Extracting peak value from sEMG envelope signal

Identification and calculation of sEMG envelope signal

Calculating muscle recruitment velocity

Calculating MVC

Finish

**Appendix 6: results of normal distribution**

| MVC | Weeks | Control group | R.I.C.E. treatment group | IT group |
| --- | --- | --- | --- | --- |
| Biceps brachii | 1 | 0.129 | 0.200 | 0.200 |
|  | 5 | 0.200 | 0.200 | 0.200 |
|  | 10 | 0.200 | 0.079 | 0.200 |
| Triceps brachii | 1 | 0.079 | 0.200 | 0.121 |
|  | 5 | 0.200 | 0.054 | 0.200 |
|  | 10 | 0.200 | 0.184 | 0.200 |
| Brachioradialis | 1 | 0.200 | 0.200 | 0.200 |
|  | 5 | 0.200 | 0.200 | 0.200 |
|  | 10 | 0.200 | 0.118 | 0.200 |
| Forearm flexor & extensor | 1 | 0.200 | 0.147 | 0.078 |
|  | 5 | 0.030 | 0.200 | 0.200 |
|  | 10 | 0.200 | 0.200 | 0.200 |
| Average (4 muscles) | 1 | 0.146 | 0.050 | 0.109 |
|  | 5 | 0.086 | 0.108 | 0.129 |
|  | 10 | 0.200 | 0.062 | 0.156 |
